# HLA-B, HLA-C and KIR improve the predictive value of IFNL3 for Hepatitis C spontaneous clearance

**DOI:** 10.1038/s41598-017-17531-7

**Published:** 2018-01-12

**Authors:** Mario Frias, Antonio Rivero-Juárez, Diego Rodriguez-Cano, Ángela Camacho, Pedro López-López, María Ángeles Risalde, Bárbara Manzanares-Martín, Teresa Brieva, Isabel Machuca, Antonio Rivero

**Affiliations:** 1 Infectious Diseases Unit, Instituto Maimónides de Investigación Biomédica de Córdoba (IMIBIC), Hospital Universitario Reina Sofía de Córdoba, Córdoba, Spain; 2 Immunology Unit, Instituto Maimónides de Investigación Biomédica de Córdoba (IMIBIC), Hospital Universitario Reina Sofía de Córdoba, Córdoba, Spain

## Abstract

IFNL3 is the strongest predictor of spontaneous resolution (SR) of hepatitis C virus (HCV), however, consideration of IFNL3 genotype alone is of limited clinical value for the prediction of SR or chronic HCV infection. The objective of this study was to analyze the impact of HLA-B, HLA-C and KIRs on SR, as well as their additive effects on the predictive value of the IFNL3 genotype. We conducted a retrospective study of HIV patients that included both SR and chronic HCV patients. In our study, 61.6% of patients with IFNL3 CC achieved SR, and 81.5% with non-CC genotypes did not achieve SR. HLA-B*44, HLA-C*12, and KIR3DS1 were identified as predictive factors for SR, with percentages of 77.4%, 85.7% and 86.2%, respectively, for patients who did not experience SR. The presence of at least one of these three markers, defined as a genetically unfavorable profile (GUP), combined with the IFNL3 non-CC genotype showed a value of 100% for non-SR. The absence of the three markers, defined as a genetically favorable profile (GFP), in addition to the IFNL3 CC genotype showed a percentage of 74.1% for SR. The combination of these markers in addition to the IFNL3 genotype improves the predictive value of IFNL3 for SR of acute HCV infection in HIV patients, which would be clinically valuable.

## Introduction

After primary infection, hepatitis C virus (HCV) causes acute hepatitis that progresses in most cases to a chronic infection, characterized by the gradual advanced of liver fibrosis, cirrhosis, and hepatocellular carcinoma^1^. A percentage of patients, varying between 10 and 40%, spontaneously resolve acute HCV infection^2^. The identification of markers for predicting spontaneous resolution (SR) or chronic infection could therefore have a clinical impact in determining whether therapy should be implemented or deferred.

Several factors favoring SR have been identified, such as gender, age and race, but as this event is mediated by the host immunological system, immune-related factors have been shown to have the greatest impact on the outcome of HCV acute infection. Several polymorphisms located on interferon lambda-3 (IFNL3, also known as IL28B) have been shown to have a strong association with SR^3–7^. Because of the relatively high likelihood of self-limited infection, clinical guidelines have recognized IFNL3 as a predictive factor during acute HCV to ascertain whether to implement or defer therapy^8^. Nonetheless, a significant number of patients do not experience SR despite a favorable IFNL3 genotype, and there are also patients with unfavorable non-CC genotypes whose HCV infections are self-limiting^3–5^. Consideration of IFNL3 alone is therefore of limited value for determining which patients will resolve HCV spontaneously. The identification of additional markers could help to improve the predictive value of IFNL3 for acute HCV patients, which would be clinically valuable.

Human leukocyte antigen (HLA) class I and killer cell immunoglobulin-like receptors (KIR) modulate the function of immune cells such as NK and T cells. These immunological markers play a major role in the regulation of immune responses against HCV infection^9–11^. A variety of different HLA alleles and KIR genes therefore could influence viral persistence^12–16^. Few studies however have considered the IFNL3 genotype in association with these immunological markers as predictive factors for the SR of HCV infection.

Therefore, the aim of our study was to analyze the impacts of several immunological factors, such as HLA-B, HLA-C and KIRs, on SR, as well as their additive effect on the IFNL3 genotype for this outcome.

## Results

### Baseline patient characteristics

Fifty-seven HIV-infected patients who experienced SR and 81 patients who developed chronic HCV infections were included in this retrospective study. All patients included were Caucasian. HCV genotype was not available for 89.7% of patients who achieved SR. The baseline characteristics of these patients are shown in Table 1. Due to the retrospective nature of the study, we were unable to ascertain the exact age when SR was reached or exclude the possibility that some individuals were exposed multiple times. The distribution of the HLA-B, HLA-C and KIR genotypes is shown in Supplementary Tables S1, S2, and S3, respectively.

**Table 1:** Major baseline patient characteristics.

Clinical characteristics	Condition	SR	Non-SR	*p*	OR for SR (95% CI)	OR for non-SR (95% CI)
Age (median = 50 years), n (%)	<50 years	33 (47.8)	36 (52.2)	0.120	1.72 (0.86 to 3.43)	1
	≥50 years	24 (34.8)	45 (65.2)		1	0.58 (0.29 to 1.16)
Sex, n (%)	Male	38 (36.9)	65 (63.1)	0.071	0.49 (0.22 to 1.08)	1
	Female	19 (54.3)	16 (45.7)		1	2.03 (0.93 to 4.46)
CD4^+^ (median = 551 cells/mL), n (%)	<551 cells/mL	32 (46.4)	37 (53.6)	0.226	1.52 (0.77 to 3.03)	1
	≥551 cells/mL	25 (36.2)	44 (63.8)		1	0.66 (0.77 to 3.03)
HIV viral load, n (%)	Undetectable	52 (41.6)	73 (58.4)	0.827	1.14 (0.35 to 4.02)	1
	Detectable	5 (38.5)	8 (61.5)		1	0.88 (0.25 to 2.87)
Use of ART, n (%)	Yes	55 (40.4)	81 (59.6)	0.169	0 (0 to 2.42)	1
	No	2 (100)	0		1	Infinity (0.41 to infinity)
AIDS defining criteria in the past, n (%)^a^	Yes	16 (45.7)	19 (54.3)	0.540	1.27 (0.58 to 2.77)	1
	No	41 (39.8)	62 (60.2)		1	0.79 (0.36 to 1.73)

Abbreviations: spontaneous resolution (SR); non-spontaneous resolution (non-SR); 95% confidence interval (95% CI); odds ratio (OR); n (number of cases); human immunodeficiency virus (HIV); antiretroviral therapy (ART); and acquire immunodeficiency syndrome (AIDS), ^a^Centers for Disease Control and Prevention: revised surveillance case definitions for HIV infection among adults, adolescents, and children aged <18 months and for HIV infection and AIDS among children aged 18 months to <13 years, United States, 2008.

### Factors associated with SR and non-SR

Forty-five of 73 patients (61.6%) with the IFNL3 CC genotype experienced SR (OR = 7.1 [95% CI: 3.23 to 15.81]). Among patients with the IFNL3 non-CC genotype, 53 of 65 (81.5%) did not experience SR (OR = 0.14 [95% CI: 0.06 to 0.33]) (Table 2). When the HLA-B alleles were analyzed, 41 of 94 (43.6%) patients with absence of HLA-B*44 achieved SR (OR = 2.65 [95% CI: 1.06 to 7.17) and 24 of 31 (77.4%) patients with presence of HLA-B*44 did not achieve SR (OR = 0.38 [95% CI: 0.14 to 0.95) (Table 2 and Supplementary Table S1). With respect to the HLA-C alleles, in the absence of HLA-C*07, 39 of 84 (46.4%) patients reached SR (OR = 2.71 [95% CI: 1.10 to 7]), and when it was present, 25 of 33 (75.8%) did not reach SR (OR = 0.37 [95% CI: 0.14 to 0.91]) (Table 2 and Supplementary Table S2). In the absence of HLA-C*12, 44 of 96 (45.8%) patients achieved SR (OR = 5.08 [95% CI: 1.49 to 22.6]) and when it was present, 18 of 21 (85.7%) patients did not achieve SR (OR = 0.20 [95% CI: 0.04 to 0.67]) (Table 2 and Supplementary Table S2). When the KIR genes were analyzed, 30 of 81 (47.6%) patients with absence of KIR3DS1 experienced SR (OR = 5.68 [95% CI: 1.94 to 20.05]), and with its presence, 25 of 29 (86.2%) did not experience SR (OR = 0.18 [95% CI: 0.05 to 0.52]) (Table 2 and Supplementary Table S3). In the multivariate analysis, the IFNL3 CC genotype, the presence of HLA-B*44, the presence of HLA-C*12, and the presence of KIR3DS1 were shown to be independent factors associated with SR (Table 3).

**Table 2:** Percentages, p-values and odds ratio for Spontaneous Resolution (SR) and non-SR.

Factor	Condition	SR; n (%)	non-SR; n (%)	p-value	OR for SR (95%CI)	OR for non-SR (95% CI)
IFNL3	CC	45 (61.6)	28 (38.4)	<0.001	7.1 (3.23 to 15.81)	1
	Non-CC	12 (18.5)	53 (81.5)		1	0.14 (0.06 to 0.33)
HLA-B*44	Absence	41 (43.6)	53 (56.4)	0.037	2.65 (1.06 to 7.17)	1
	Presence	7 (22.6)	24 (77.4)		1	0.38 (0.14 to 0.95)
HLA-C*07	Absence	39 (46.4)	45 (53.6)	0.028	2.71 (1.10 to 7)	1
	Presence	8 (24.2)	25 (75.8)		1	0.37 (0.14 to 0.91)
HLA-C*12	Absence	44 (45.8)	52 (54.2)	0.008	5.08 (1.49 to 22.6)	1
	Presence	3 (14.3)	18 (85.7)		1	0.20 (0.04 to 0.67)
KIR3DS1	Absence	50 (47.6)	55 (52.4)	<0.001	5.68 (1.94 to 20.05)	1
	Presence	4 (13.8)	25 (86.2)		1	0.18 (0.05 to 0.52)

Abbreviations: spontaneous resolution (SR); non-spontaneous resolution (non-SR); n (number of cases); 95% confidence interval (95% CI); odds ratio (OR).

**Table 3:** Multivariate analysis^1^ for SR.

Factor	Condition	*p*	OR for SR (95% CI)
IFNL3	Non CC	**0.001**	1
	CC		6.43 (2.10 to 19.08)
HLA-B44	Absence	**0.006**	1
	Presence		0.18 (0.05 to 0.62)
HLA-C07	Absence	0.647	1
	Presence		0.75 (0.20 to 2.59)
HLA-C12	Absence	**0.029**	1
	Presence		0.17 (0.03 to 1.06)
KIR3DS1	Absence	**0.001**	1
	Presence		0.09 (0.01 to 0.50)
Gender	Male	0.519	1
	Female		1.50 (0.48 to 4.63)
Age	<50 years	0.929	1
	≥50 years		0.94 (0.31 to 2.80)

^1^No interactions were found between the factors included in the model (IFNL3* HLA-B44, p = 0.999; IFNL3*HLA-C12, p = 0.998; IFNL3*KIR3DS1, p = 0.998; HLA-B44*HLA-C12, p = 0.324; HLA-B44*KIR3DS1, p = 0.999; HLA-C12*KIR3DS1, p = 0.999). Abbreviations: spontaneous resolution (SR); non-spontaneous resolution (non-SR); n (number of cases); 95% confidence interval (95% CI); odds ratio (OR).

### Combination of HLA-B*44, HLA-C*12, KIR3DS1 and IFNL3 in the prediction of SR

**Figure 1:**
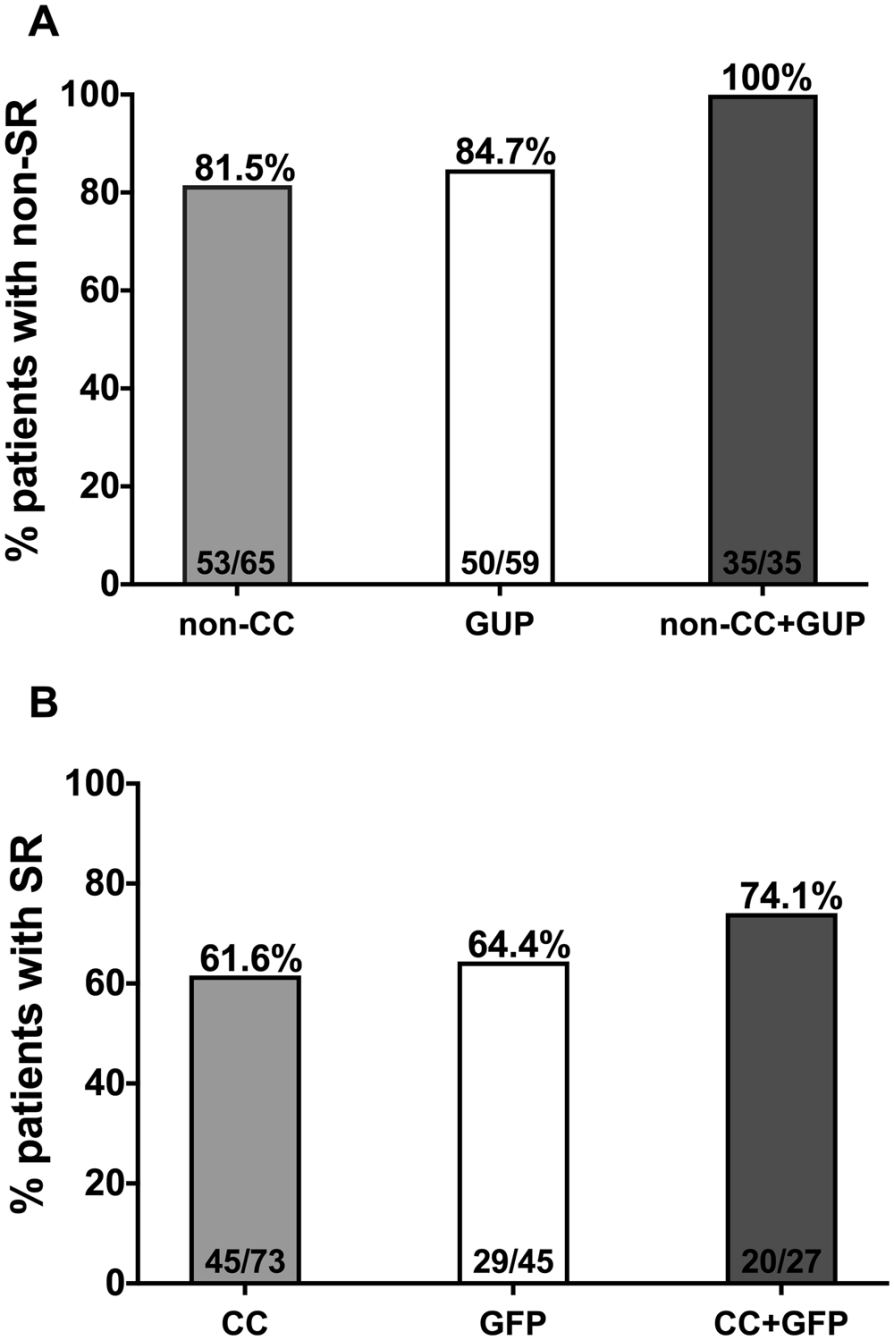
Percentage of patients who did not achieve SR according to IFNL3 non-CC and IFNL3 non-CC plus genetically unfavorable profile (GUP) (**A**). Percentage of patients who achieved SR according to IFNL3 CC and IFNL3 CC plus genetically favorable profile (GFP) for SR (**B**). GFP is defined as absence of HLA-B*44, HLA-C*12 and KIR3DS1. GUP is defined as presence of at least one gene of HLA-B*44, HLA-C*12 and KIR3DS1.

The IFNL3 CC and non-CC genotypes, the presence/absence of the KIR3DS1 genotype, the presence/absence of the HLA-B*44 allele and the presence/absence of the HLA-C*12 allele were analyzed with regard to their impact on SR (Supplementary Table S4). The combination of the three favorable factors (absence of HLA-B*44, HLA-C*12 and KIR3DS1 genes) was defined as a genetically favorable profile for SR (GFP), whereas the presence of one of the three unfavorable factors (HLA-B*44, HLA-C*12 or KIR3DS1) was defined as a genetically unfavorable profile (GUP) for SR. Among those patients with a GFP, 29 of 45 (64.4%) experienced SR (OR = 10.07 [95% CI: 3.92 to 26.19]), whereas 50 of 59 (84.7%) patients with the GUP did not experience SR (OR = 0.10 [95% CI: 0.04 to 0.26]). When the involvement of these two gene profiles was analyzed for SR together with IFNL3, 35 of 35 (100%) patients with the GUP and the IFNL3 non-CC genotype (Fig. 1A) did not achieve SR (OR = 0 [95% CI: 0 to 0.13]. Additionally, 20 of 27 (74.1%) patients with the GFP and with the IFNL3 CC genotype (Fig. 1B) experienced SR (OR = 6.34 [95% CI: 2.56 to 16.03]).

### Area under the receiver operating characteristic (AUROC) for SR

The AUROC curve for the IFNL3 CC genotype was 0.715 (0.612–0.818) for SR. The AUROC curve for the GFP was 0.760 (0.662–0.859) for SR. When the IFNL3 CC genotype and the GFP were analyzed together, the AUROC curve was 0.710 (0.599–0.82). Similarly, the AUROC curve for the IFNL3 non-CC genotype was 0.715 (0.612–0.818) for non-SR. The AUROC curve for the GUP was 0.760 (0.662–0.859) for non-SR. When the IFNL3 non-CC genotype and the GUP were analyzed together, the AUROC value was 0.765 (0.676–0.853).

## Discussion

The association of the IFNL3 genotype with SR has been widely described and supports the importance of interferon lambda and innate immunity in the outcome of HCV infection^3–7^. The first evidence was reported by Thomas *et al*., who showed that patients with the IFNL3 CC genotype had a higher rate of SR (53%) than those with the T allele (28%)^3^. Tillmann *et al*. found that 64% patients with the CC genotype experienced SR, while the rate of SR for patients with non-CC genotypes was 19%^4^. Similarly, Knapp *et al*., found that the frequency of the IFNL3 CC genotype was significantly higher in patients with SR compared to those who were chronically infected (69.7% and 43.6%, respectively). In contrast, the percentage of IFNL3 non-CC genotype was lower in patients with SR than in those with chronic HCV infections (30.3% and 56.4%, respectively)^5^. Despite numerous studies showing the IFNL3 CC genotype to be strongly associated with the natural control of HCV infection, there are a significant proportion of patients are misclassified according to this SNP as 30–40% of patients with an IFNL3 CC genotype will develop a chronic HCV infection and approximately 20–40% of patients with an IFNL3 non-CC genotype will experience SR. Likewise, the favorable IFNL3 genotype in our study was found in 61.6% of patients with self-limiting acute HCV infection, and 38.4% of patients with an IFNL3 CC genotype developed a chronic HCV infection. For this reason, the consideration of only the IFNL3 genotype is insufficient for clinical decision making. Therefore, other factors are needed to more widely predict which patients will have SR and which patients will not.

Major histocompatibility complex (MHC) class I molecules have an important role in the outcome of several inflammatory and infectious diseases^9–11^. Specific HLAs can increase or decrease the elimination of HCV-infected cells^12–14^. In the context of SR, two HLA-B alleles, HLA-B*27 and HLA-B*57, have been previously associated with self-limiting infections. First, two studies associated HLA-B*27 with SR^17,18^, although the association was limited only to patients with an HCV genotype 1, because no association was observed with other genotypes^19^. Second, HLA-B*57 was also associated with SR in a several studies^20,21^, though this association was not found in another study^22^. HLA-C alleles have also been shown to affect the outcome of HCV infection. Two studies found HLA-C*05 to be associated with the viral persistence of HCV^20,23^. In our study, the presence of HLA-B*44 and HLA-C*12 were independent factors associated with HCV chronicity, showing higher frequencies among patients who developed chronic HCV infections (HLA-B*44: 77.4%; HLA-C*12: 85.7%).

With respect to KIR genes and their associations with HCV chronicity, Dring *et al*. showed that the frequency of the KIR2DS3 receptor was significantly increased in patients who had chronic HCV infections^15^. Similarly, a study by De Re *et al*. also found that KIR2DS3 was the principal gene related to HCV viral persistence^16^. A study of risk for mother-to-child transmission of HCV found that KIR3DS1 was associated with development of chronic HCV infection^24^. Specifically, Ruiz-Extremera *et al*. found that the children of mothers with KIR3DS1 had a higher risk of HCV chronicity than the children of mothers without this receptor (75% and 19%, respectively)^24^. In our study, the presence of the KIR3DS1 receptor was also linked with chronic HCV infection, showing a higher frequency in patients who had chronic HCV infections (86.2%).

The combination of several immunological markers for the prediction of SR has been little studied^18,25–28^. Mangia *et al*. observed that the IFNL3 CC genotype in combination with DBQ1*0301 increased the accuracy of SR prediction from 63% to 69%^25^. Likewise, Duggal *et al*. found that the combination of DQB1*03:01 with the IFNL3 genotype could increase the positive predictive value for SR from 45% to 60%^26^. In contrast, Huang *et al*. found that two HLA alleles (A*02 and DRB1*1) together with IFNL3 did not significantly improve the prediction of the probability of SR (57.0% to 57.8%)^27^. In our study, we found that the combination of three markers, HLA-B*44, HLA-C*12 and KIR3DS1, improved the predictive value of IFNL3 for SR. In particular, no patient with the IFNL3 non-CC genotype (0%) achieved SR when they also had a GUP. Consequently, the consideration of these four genetic markers identified a large population of patients with low probabilities of achieving SR. Moreover, the percentage of SR in patients with an IFNL3 CC genotype significantly increased when a GFP is also considered (61% to 74.1%). In contrast, the likelihood of SR among patients with an IFNL3 CC genotype significantly dropped from 61% to 30.4% in combination with a GUP. These findings could significantly improve the predictive accuracy of IFNL3 for determining the probability of achieving SR.

In patients with acute HCV infection, a dilemma exists regarding whether to delay or initiate HCV treatment while waiting to see if SR occurs. It is beneficial to delay treatment based on the likelihood of SR as it reduces unnecessary therapy in closely monitored populations^28^. Current HCV guidelines identify IFNL3 as an immunological marker for clinical decision making^8^. Nonetheless, by following this association, our study showed that almost 40% of patients were mismatched according to their IFNL3 CC genotype. Our results suggest that determining HLA-B*44, HLA-C*12 and KIR3DS1 would improve the predictive value of IFNL3 for clinical decision making in this setting. The real impact of the combination of these markers would be to enhance the prediction of SR in patients who have all of the unfavorable markers (IFNL3 non-CC genotype and a GUP), because the likelihood that such patients will experience SR is low and, consequently, they could benefit from immediate treatment implementation. In addition, in these patients, taking time to wait for SR would be unnecessary, thus significantly reducing the risk of HCV transmission. In contrast, in patients who have favorable markers (IFNL3 CC genotype and a GFP), HCV therapy could be deferred in the short-term because of the high probability that they will experience SR. Nevertheless, before this combination of markers can be implemented in clinical practice, the confirmation of our results in a validation cohort is mandatory.

Our study has several limitations that should be noted. First, this study included only HIV/HCV co-infected patients, and therefore, these results cannot be extrapolated to HCV mono-infected individuals. Second, HLA-A and all HLA-II genotyping was not available in this study; therefore, we do not know the role of all classic HLA class I and HLA class II molecules. Third, this study included a relatively small number of patients. Thus, we may not have enough statistical power to detect associations with other HLA alleles and KIR genotypes. Finally, due to the retrospective nature of the study, age at HCV infection, multiple exposure risk and viral genotype were unknown. Consequently, these factors, which have been associated with SR in other studies^29,30^, were not analyzed in this study.

In conclusion, our study identifies immunological factors, including the HLA-B*44 and HLA-C*12 alleles and the KIR3DS1 genotype, as independent factors associated with HCV chronicity in HIV patients. Furthermore, these factors improved the predictive capacity of IFNL3 for the outcome of acute HCV infection. A validation cohort is needed to confirm these results.

## Material and Methods

### Patients

HIV patients from the Infectious Diseases Unit at the Hospital Reina Sofía de Córdoba (Spain) were included in this retrospective study between 2013 and 2016. The patients were included in two groups. The first group corresponded to SR patients and all those who fulfilled the following criteria were included: i) positive HCV antibodies confirmed by recombinant immunoblot assay and negative HCV RNA without the patient ever having received anti-HCV therapy, or ii) HCV RNA detected only in the first 6 months after HCV seroconversion. Patients who were active intravenous drug users (IDU) were excluded from the study. The second group comprised patients with chronic HCV infection, and the criteria for inclusion were: i) persistent detection of HCV RNA for more than 6 months, and ii) HCV treatment-inexperienced patients.

### Variable collection and definition

The variables collected included the genotypes of IFNL3, KIR, HLA-B, and HLA-C; age; gender; baseline CD4^+^ cell count (cells/mL); HIV viral load; antiretroviral therapy (ART) usage; acquired immunodeficiency syndrome (AIDS) condition; and HCV genotype.

IFNL3 SNP rs129679860 was genotyped using a custom TAQMAN assay (Applied Biosystems, Foster City, California, USA), according to the manufacturer’s instructions. The IFNL3 genotype was defined as CC or non-CC (TT/CT). KIR genotyping was performed using sequence-specific primers to detect the presence of 16 different KIR genes as described previously by Gomez-Lozano *et al*.^31^. This method provided a high degree of resolution since each primer pair identifies two linked, cis-located polymorphic sites (Invitrogen, Thermo Fisher Scientific, Massachusetts, USA). Only KIR genes with frequencies greater than 5% were included in the analysis. HLA-B genotyping was performed with the INNO-LIPA HLA-B Multiplex kit (Innogenetics N.V., Ghent, Belgium), using HLA-B multiplex primers for amplification of the second and fourth exon of the HLA-B locus. This technique was based on the PCR-SSO reverse method. HLA-C genotyping was performed according to the method previously described by Turner *et al*. for amplification of the second and third exon of the HLA-C locus^32^. Only HLA-B and HLA-C alleles with frequencies greater than 5% were included in the analysis.

### Statistical Analysis

Categorical variables are expressed as numbers of cases and percentages. Frequencies were compared using the χ^2^ or Fisher’s exact tests to analyze associations for SR or chronic HCV. Significance was set at a *p* value of less than 0.05. A logistic regression model for predicting SR was performed and tested by the bootstrap method (1,000 replicates). The area under the receiver operating characteristic (AUROC) curve was used to evaluate sensitivity and specificity for IFNL3 and additional genetic markers, in accordance with the explanation and elaboration of studies of diagnostic accuracy described by Bossuyt *et al*.^33^. These analyses were performed using the SPSS statistical software package, version 18.0 (IBM Corporation, Somers, NY, USA) and GraphPad Prism version 6 (Mac OS X version; GraphPad Software, San Diego, CA, USA).

### Ethical statement

This study was designed and performed according to the Helsinki Declaration. All patients signed an informed consent form that gave permission for the storage and processing of material from routine diagnostic processes at the Biobanco del Hospital Universitario Reina Sofía de Córdoba (ISCIII reference: B.0000419), which is integrated into the Biobanco del Sistema Sanitario Público de Andalucía. The CEIC (Clinical Trial and Ethical Committee) of the Hospital Universitario Reina Sofía de Córdoba approved the study protocol.

### Data availability

All data generated or analyzed during this study are included in this published article (and its Supplementary Information files).

## Electronic supplementary material

Supplementary information
