# Supplementary material for: HLA-B, HLA-C and KIR improve the predictive value of IFNL3 for Hepatitis C spontaneous clearance
Source: Sci Rep. 2018 Jan 12;8:659. doi: 10.1038/s41598-017-17531-7 (PMC5766528; doi:10.1038/s41598-017-17531-7)
Supplement: Supplementary file 1 — Supplementary information [file 41598_2017_17531_MOESM1_ESM.doc]

**HLA-B, HLA-C and KIR improve the predictive value of IFNL3 for Hepatitis C spontaneous clearance**

**Authors:** Mario Frias, Antonio Rivero-Juárez, Diego Rodriguez-Cano, Ángela Camacho, Pedro López-López, María Ángeles Risalde, Bárbara Manzanares-Martín, Teresa Brieva, Isabel Machuca and Antonio Rivero

**Table S1. Bivariate analysis between HLA-B and SR vs. non-SR.**

| **HLA-B^1^** | **Condition** | **SR; n (%)** | **non-SR; n (%)** | ***p*** | **OR for SR (95% CI)** | **OR for non-SR (95% CI)** |
| --- | --- | --- | --- | --- | --- | --- |
| *07 | Absence | 38 (40.9) | 55 (59.1) | 0.335 | 1.52 (0.65 to 3.69) | 1 |
|  | Presence | 10 (31.2) | 22 (68.8) |  | 1 | 0.66 (0.27 to 1.54) |
| *14 | Absence | 37 (37) | 63 (63) | 0.520 | 0.75 (0.31 to 1.87) | 1 |
|  | Presence | 11 (44) | 14 (56) |  | 1 | 1.34 (0.54 to 3.28) |
| *15 | Absence | 46 (39) | 72 (61) | 0.706 | 1.60 (0.30 to 12.27) | 1 |
|  | Presence | 2 (28.6) | 5 (71.4) |  | 1 | 0.63 (0.08 to 3.33) |
| *18 | Absence | 43 (39.1) | 67 (60.9) | 0.667 | 1.28 (0.41 to 4.40) | 1 |
|  | Presence | 5 (33.3) | 10 (66.7) |  | 1 | 0.78 (0.15 to 3.31) |
| *27 | Absence | 45 (38.8) | 71 (61.2) | 0.999 | 1.27 (0.30 to 6.47) | 1 |
|  | Presence | 3 (33.3) | 6 (66.7) |  | 1 | 0.79 (0.12 to 3.93) |
| *35 | Absence | 41 (40.2) | 61 (59.8) | 0.385 | 1.54 (0.59 to 4.30) | 1 |
|  | Presence | 7 (30.4) | 16 (69.6) |  | 1 | 0.65 (0.23 to 1.71) |
| *38 | Absence | 47 (40.5) | 69 (59.5) | 0.151 | 5.45 (0.82 to 124.3) | 1 |
|  | Presence | 1 (11.1) | 8 (88.9) |  | 1 | 0.18 (0.01 to 1.21) |
| *40 | Absence | 44 (39.3) | 68 (60.7) | 0.765 | 1.46 (0.43 to 5.72) | 1 |
|  | Presence | 4 (30.8) | 9 (69.2) |  | 1 | 0.69 (0.17 to 2.35) |
| *44 | Absence | 41 (43.6) | 53 (56.4) | **0.033** | 2.65 (1.06 to 7.17) | 1 |
|  | Presence | 7 (22.6) | 24 (77.4) |  | 1 | 0.38 (0.14 to 0.95) |
| *49 | Absence | 44 (37.9) | 72 (62.1) | 0.732 | 0.76 (0.18 to 3.36) | 1 |
|  | Presence | 4 (44.4) | 5 (55.6) |  | 1 | 1.31 (0.30 to 5.42) |
| *51 | Absence | 41 (37.3) | 69 (62.7) | 0.483 | 0.68 (0.22 to 2.11) | 1 |
|  | Presence | 7 (46.7) | 8 (53.3) |  | 1 | 1.47 (0.47 to 4.47) |
| *52 | Absence | 42 (38.2) | 68 (61.8) | 0.892 | 0.93 (0.30 to 2.98) | 1 |
|  | Presence | 6 (40) | 9 (60) |  | 1 | 1.08 (0.34 to 3.29) |

Abbreviations: spontaneous resolution (SR); non-spontaneous resolution (non-SR); n (number of cases); 95% confidence interval (95% CI); odds ratio (OR).

^1^Frequencies of the B*08, B*13, B*37, B*39, B*41, B*45, B*50, B*57 and B*58 alleles are not shown (all of these frequencies were lower than 5%).

**Table S2. Bivariate analysis between HLA-C and SR vs. non-SR.**

| **HLA-C^1^** | **Condition** | **SR; n (%)** | **non-SR; n (%)** | ***p*** | **OR for SR (95% CI)** | **OR for non-SR (95% CI)** |
| --- | --- | --- | --- | --- | --- | --- |
| *02 | Absence | 44 (41.5) | 62 (58.5) | 0.522 | 1.89 (0.49 to 9.2) | 1 |
|  | Presence | 3 (27.3) | 8 (72.7) |  | 1 | 0.53 (0.11 to 2.06) |
| *03 | Absence | 44 (41.5) | 62 (58.5) | 0.522 | 1.89 (0.49 to 9.2) | 1 |
|  | Presence | 3 (27.3) | 8 (72.7) |  | 1 | 0.53 (0.11 to 2.06) |
| *04 | Absence | 38 (41.3) | 54 (58.7) | 0.818 | 1.25 (0.5 to 3.25) | 1 |
|  | Presence | 9 (36) | 16 (64) |  | 1 | 0.80 (0.31 to 2) |
| *05 | Absence | 35 (38.5) | 56 (61.5) | 0.480 | 0.73 (0.3 to 1.8) | 1 |
|  | Presence | 12 (46.2) | 14 (53.8) |  | 1 | 1.37 (0.56 to 3.33) |
| *06 | Absence | 45 (41.3) | 64 (58.7) | 0.472 | 2.11 (0.42 to 15.69) | 1 |
|  | Presence | 2 (25) | 6 (75) |  | 1 | 0.47 (0.06 to 2.37) |
| *07 | Absence | 39 (46.4) | 45 (53.6) | **0.028** | 2.71 (1.1 to 7) | 1 |
|  | Presence | 8 (24.2) | 25 (75.8) |  | 1 | 0.37 (0.14 to 0.91) |
| *08 | Absence | 38 (39.6) | 58 (60.4) | 0.782 | 0.87 (0.33 to 2.35) | 1 |
|  | Presence | 9 (42.9) | 12 (57.1) |  | 1 | 1.14 (0.42 to 3.01) |
| *12 | Absence | 44 (45.8) | 52 (54.2) | **0.008** | 5.08 (1.49 to 22.6) | 1 |
|  | Presence | 3 (14.3) | 18 (85.7) |  | 1 | 0.20 (0.04 to 0.67) |
| *15 | Absence | 40 (38.1) | 65 (61.9) | 0.219 | 0.44 (0.12 to 1.53) | 1 |
|  | Presence | 7 (58.3) | 5 (41.7) |  | 1 | 2.27 (0.65 to 8.27) |
| *16 | Absence | 41 (40.2) | 61 (59.8) | 0.988 | 1.01 (0.33 to 3.26) | 1 |
|  | Presence | 6 (40) | 9 (60) |  | 1 | 0.99 (0.31 to 3.04) |

Abbreviations: spontaneous resolution (SR); non-spontaneous resolution (non-SR); n (number of cases); 95% confidence interval (95% CI); odds ratio (OR).

^1^Frequencies of the C*01 and C*14 alleles are not shown (both frequencies were lower than 5%).

**Table S3. Bivariate analysis between KIR genotypes and SR vs. non-SR.**

| **KIR^1^** | **Condition** | **SR; n (%)** | **non-SR; n (%)** | ***p*** | **OR for SR (95% CI)** | **OR for non-SR (95% CI)** |
| --- | --- | --- | --- | --- | --- | --- |
| 2DS2 | Absence | 30 (46.2) | 35 (53.8) | 0.202 | 1.57 (0.78 to 3.17) | 1 |
|  | Presence | 24 (35.3) | 44 (64.7) |  | 1 | 0.64 (0.32 to 1.28) |
| 2DL2 | Absence | 18 (35.3) | 33 (64.7) | 0.397 | 0.73 (0.35 to 1.51) | 1 |
|  | Presence | 35 (42.7) | 47 (57.3) |  | 1 | 1.37 (0.66 to 2.84) |
| 2DL3 | Absence | 3 (33.3) | 6 (66.7) | 0.999 | 0.74 (0.15 to 3.14) | 1 |
|  | Presence | 49 (40.2) | 73 (59.8) |  | 1 | 1.34 (0.32 to 6.83) |
| 2DL5 | Absence | 23 (37.7) | 38 (62.3) | 0.531 | 0.80 (0.4 to 1.61) | 1 |
|  | Presence | 31 (43.1) | 41 (56.9) |  | 1 | 1.25 (0.62 to 2.53) |
| 2DS3 | Absence | 22 (31) | 49 (69) | 0.071 | 0.51 (0.24 to 1.07) | 1 |
|  | Presence | 24 (47.1) | 27 (52.9) |  | 1 | 1.98 (0.93 to 4.20) |
| 2DS5 | Absence | 41 (43.2) | 54 (56.8) | 0.188 | 1.73 (0.76 to 4.02) | 1 |
|  | Presence | 11 (30.6) | 25 (69.4) |  | 1 | 0.58 (0.25 to 1.31) |
| 2DP1 | Absence | 1 (20) | 4 (80) | 0.648 | 0.35 (0.01 to 2.93) | 1 |
|  | Presence | 53 (41.4) | 75 (58.6) |  | 1 | 2.83 (0.34 to 71.18) |
| 2DL1 | Absence | 4 (44.4) | 5 (55.6) | 0.999 | 1.15 (0.26 to 4.73) | 1 |
|  | Presence | 53 (41.1) | 76 (58.9) |  | 1 | 0.87 (0.21 to 3.81) |
| 3DL1 | Absence | 9 (60) | 6 (40) | 0.119 | 2.38 (0.78 to 7.54) | 1 |
|  | Presence | 48 (39) | 75 (61) |  | 1 | 0.43 (0.13 to 1.30) |
| 3DS1 | Absence | 50 (47.6) | 55 (52.4) | **0.001** | 5.68 (1.94 to 20.05) | 1 |
|  | Presence | 4 (13.8) | 25 (86.2) |  | 1 | 0.18 (0.05 to 0.52) |
| 2DS1 | Absence | 30 (37) | 51 (63) | 0.296 | 0.69 (0.34 to 1.4) | 1 |
|  | Presence | 24 (46.2) | 28 (53.8) |  | 1 | 1.46 (0.71 to 2.97) |
| 2DS4 | Absence | 40 (42.6) | 54 (57.4) | 0.376 | 1.42 (0.65 to 3.19) | 1 |
|  | Presence | 13 (34.2) | 25 (65.8) |  | 1 | 0.70 (0.31 to 1.54) |
| 1D | Absence | 12 (50) | 12 (50) | 0.300 | 1.60 (0.64 to 3.94) | 1 |
|  | Presence | 42 (38.5) | 67 (61.5) |  | 1 | 0.63 (0.25 to 1.56) |

Abbreviations: spontaneous resolution (SR); non-spontaneous resolution (non-SR); n (number of cases); 95% confidence interval (95% CI); odds ratio (OR).

^1^The framework genes KIR2DL4, 3DL2, 3DL3 and 3DP1 were present in all of the patients.

**Table S4. Impact of HLA-B, HLA-C and KIR3DS1 on the predictive values of IFNL3**

| **IFNL3** | **HLA-B*44** | **HLA-C*12** | **KIR3DS1** | ***n^1^*** | ***SR^2^*** | ***% SR*** | ***% non-SR*** |
| --- | --- | --- | --- | --- | --- | --- | --- |
| CC | Presence | Presence | Presence | 1 | 0 | 0% | 100% |
|  | Presence | Absence | Presence | 2 | 0 | 0% | 100% |
|  | Presence | Absence | Absence | 11 | 5 | 45.4% | 54.5% |
|  | Presence | Presence | Absence | 2 | 1 | 50% | 50% |
|  | Absence | Presence | Presence | 1 | 0 | 0% | 100% |
|  | Absence | Presence | Absence | 3 | 1 | 33% | 66.7% |
|  | Absence | Absence | Presence | 4 | 2 | 50% | 50% |
|  | Absence | Absence | Absence | 27 | 20 | 74.1% | 25.9% |
| Non-CC | Presence | Presence | Presence | 1 | 0 | 0% | 100% |
|  | Presence | Absence | Presence | 0 | 0 | 0% | 0% |
|  | Presence | Absence | Absence | 9 | 0 | 0% | 100% |
|  | Presence | Presence | Absence | 0 | 0 | 0% | 0% |
|  | Absence | Presence | Presence | 5 | 0 | 0% | 100% |
|  | Absence | Presence | Absence | 7 | 0 | 0% | 100% |
|  | Absence | Absence | Presence | 13 | 0 | 0% | 100% |
|  | Absence | Absence | Absence | 18 | 9 | 50% | 50% |

Abbreviations: spontaneous resolution (SR); non-spontaneous resolution (non-SR); n (number of cases).

^1^HLA-B, HLA-C and KIR genotypes were not available for 14, 20, and 4 patients, respectively.

^2^Number of patients who achieved spontaneous resolution of an HCV infection
